# Professionals’ self-rated quality of care and its relation to competence, national guidelines and policies - a cross-sectional study among Finnish elderly care workers

**DOI:** 10.1186/s12913-018-3705-6

**Published:** 2018-11-26

**Authors:** Salla Lehtoaro, Kim Josefsson, Timo Sinervo

**Affiliations:** National Institute for Health and Welfare, Box 30, 00271 Helsinki, Finland

**Keywords:** Quality of care provided, Elderly care, Guidelines, Competence

## Abstract

**Background:**

In the future, elderly care workers need to have competence of various different conditions due to greater amount of multimorbid elderly. Further, knowledge of national level guidelines is important since they are closely linked to improving quality of care and implementing better practices at work places. The impact of national level guidelines on quality of care at care units is, however, not widely examined in the Finnish context. In this study, the aim was to find out if worker’s experience of his/her own competence is associated with quality of care. Secondly, we aimed to see how common is addressing national guidelines and policies at workplaces, and if they are associated with quality of care. Thirdly, we aimed to see whether there are differences between different occupational statuses in competence and addressing national guidelines and policies.

**Methods:**

Total number of respondents was 1997 from 273 different units. Xtreg procedure was used for examining the associations of age, occupational status, unit type, professional competence and addressing the guidelines and policies with quality of care.

**Results:**

Higher grade for QoC was associated with age, supervisor position, working in institutionalized care, better competence in supporting the self-determination of a person with memory disorders and falls prevention and addressing the act for elderly care and memory policy.

**Conclusion:**

This study demonstrated that national policies and guidelines are not widely addressed among Finnish elderly care workers. The study also showed that experienced competence of workers and discussion of policies and guidelines are related to quality of care. Especially competence related to memory disorders was associated with higher QoC. However, the relationship between quality of care and things influencing it seems complex and a major part of the variation in QoC remained unexplained. Although the relationships between guidelines, competences and quality of care are weak, national policies and competences seem to have impact on actual care provided. Therefore, sufficient time to address the guidelines should be provided at workplace and competences developed, which can be seen as a supervisor’s task. With knowledge about the guidelines, workers are able to change their practices at work places.

## Background

The number of elderly in Finland has increased notably during the last 50 years [1]. In 2014, the percentage of people aged 65 and over in Finland was 19,4%, this being the seventh highest percentage in The European Union [2]. Decreasing institutionalized care and increasing the amount of home care for elderly has been an aim of the Finnish government for years [3], further, home care has been seen as a priority in other countries in Europe as well [4]. ‘The Act for Elderly care’ [5] has been a guideline for Finnish elderly care for some years already. The act states that the municipality must provide the services for the elderly, the emphasis being on living at home and rehabilitation. It also underlines the quality of services and that the personnel working within the elderly services need to have for example sufficient educational qualities. Furthermore, there are other national level policies that guide the services and their provision for the elderly. The national memory policy aims to control the rising costs that the increasing amount of elderly with memory disorders would bring and to make the service chain for a person with memory disorder more unified. Further, the memory policy emphasizes competence on palliative care and the significance of rehabilitation [6]. When the amount of elderly and the proportion of people who are older than 80 years increase, so does the clinical complexity. Multimorbidity, or having 2 or more concurrent diseases, was seen to be very common among elderly people, prevalence ranging from 55% among Swedish elderly population up to 98% among Canadian elderly [7]. A large, European wide, study showed similar results; many of the elderly nursing home residents had several different medical conditions like urinary incontinence which was present among 74% of the residents. Residents also suffered from pain (36% of the residents) and impaired cognition (more than 2/3 of the residents) [8]. Also, polypharmacy is very prevalent among elderly; a study comparing the five Nordic countries found that 66% of the elderly admitted to hospital had 5 or more medications, the average number of medications per person being 6.2 [9]. Because of the complexity in the care for elderly, the workers within elderly care need to possess knowledge of various different conditions. A study from Karlsted, et al. [10] reported that nurses who work within elderly care have the need for further education especially in areas of drugs and older people, palliative and dementia care but also in rehabilitation and function disability. Bing-Jonsson et al. [11] also found in their study that there were several areas where competence should have been improved in order to secure safe care for the elderly in home care and nursing homes. When comparing those carers who had completed courses related to care with carers who had no care related education at all, results showed that those who had completed courses had higher values in factors measuring quality of care. They also perceived their workload smaller [12]. Moreover, training and an improvement in competence was seen to be associated with better patient outcomes and higher quality of care [13].

In addition, as a worker in a dynamic health care environment, one needs to be aware of the national level guidance. Guidelines are closely linked to improving quality of care and patient’s care outcomes [14, 15]. Further, they can help to develop, improve and support the evaluation of the services for the elderly [16] or improve the possibilities for elderly to participate in planning of their own care [5]. Particularly, the guidelines can provide new and better practices to be implemented at work places [16]. Guidelines can also provide specific information, like the Finnish quality recommendations for elderly care which defines the minimum carer-patient ratio in institutionalized care or those assignments that are included in the immediate patient work in home care [16]. The national memory policy provides valuable information regarding how to promote brain health emphasizing each worker’s role in the promotion [6]. Since the Finnish elderly care policy is emphasizing home care as a priority, knowledge of the factors that influence the safety of living at home is needed. The target programme for the prevention of home and leisure accident injuries suggests that each organisation involved in elderly care should have a practice for falls prevention and those workers who are doing home visits should assess the risk of falls [17]. The development of the potential of informal carers has also been a priority in Finland; National development program for informal care provides information, for instance, regarding the process of becoming an informal carer. Further, it highlights how informal care can be seen as a way of being able to live at home as long as possible [18]. Even if Finnish health care workers were found to have a positive attitude towards guidelines, they did not perceive them useful or available [19]. Further, other studies have identified several things that were seen as barriers to adhere to guidelines [20]. Similar guidelines are used in other countries too. For example, The British Geriatric Society suggests that all workers who work with elderly should use ‘Fit for Frailty’ tool to recognize frailty elderly and plan their care according to Comprehensive Geriatric Assessment [21].

The frequency of how common addressing the different national level guidance at workplaces is, has not been widely examined in the Finnish context, nor if they are associated with a workers’ self-rated quality of care that is provided in the work unit. Considering the competencies and different guidelines and policies we can see that they are interlinked and they stress similar issues regarding a worker’s knowledge and competence. The guidelines, however, are not useful, if workers do not know them. After knowing the guidelines it is possible to evaluate the working practices and to change them accordingly. Since the amount of elderly will increase in the future, competent health care professionals are required to be able to secure appropriate and equal treatment for all, in accordance to the different guidelines.

### Aim

The first aim of this study was to find out whether worker’s experience of his/her own professional competence in eight different areas affect the self-rated quality of care provided. Secondly, the study aims to find out how common is addressing different national guidelines and policies at work units and if they affect the self-rated quality of care. Thirdly, the aim was to see whether there are differences between different occupational statuses in competence and addressing national guidelines and policies.

## Methods

### Data and participants

This was a cross-sectional study and the data was gathered as a part of Personnel and work efficiency in services for older people during structural changes (HELA) study. The elderly care work units that participated to the study were selected based on their previous participation in a study regarding the Act for Elderly care and volunteered to the personnel survey also. The data consisted of 273 work units, from institutionalized care (inpatient wards and residential homes with 429 respondents) and non-institutionalized care (like assisted living facilities with 1210 respondents and home care 437 respondents) [22]. An electronic survey was sent out between December 2015 and January 2016. The survey contained mainly questions regarding working conditions, well-being, leadership and stress factors in elderly care.

### Measures

The questions considering self-assessed professional competence were of eight different areas: pain treatment, supporting the self-determination of a person with memory disorders, enhancing rehabilitation, multidimensional assessment of function, nutrition, falls prevention, palliative/end of life care and pharmacological treatment. The workers were asked to assess their own competence on a three-item scale as good (1), average (2) and need for further training (3).

Questions on whether five different national guidelines and policies had been addressed or discussed in the work place were either a yes (1) or no (0) answer. The policies and guidelines were The Act for Elderly Care, quality recommendations for elderly care, national memory policy, national development program for informal care and Target Programme for the Prevention of Home and Leisure Accident Injuries.

The quality of care provided in the work unit (QoC) was a rating scale from 4 to 10. An assessment scale from 4 to 10 is used in Finnish schools where 4 would be ‘fail’ and 10 ‘excellent’ [23]. The workers were asked to give a subjective rating as a grade for the QoC provided in their work unit.

### Statistical methods

Multilevel approach was chosen and Xtreg procedure of STATA version 13.1 was performed to examine whether age, occupational status, self-assessed competence and addressing national policies and guidelines affected the self-rated quality of care provided (QoC). The dependent variable was QoC. In the null model no other predictor variables were included since we only wanted to examine the variation of the grade for QoC and assess the intracluster correlation coefficient (ICC). Covariates in the models were age and occupational status. Gender was not used as a covariate due to an asymmetry between males (*n* = 87) and females (*n* = 1845) in the data. The reference category in the occupational status variable was (1) practical nurses or persons with similar education, the other categories were; (2) supervisors, head nurses and all others who had a supervisory position, and (3) nurses and others with bachelor’s degree. Institutionalized vs. non-institutionalized care (here on referred as ‘unit type’) was used in the model in a way that the reference group was institutionalized care including inpatient wards, residential homes and assisted living facilities with 24-h care. Non-institutionalized care was home care and ordinary assisted living facilities. Independent variables in the first model were age, occupational status, unit type and all the competence variables, eight in total. Backward elimination was used to include the most significant competence variables. In the second model, same dependent variable remained and the independent ones were age, occupational status, unit type and the most significant ones from addressing national guidelines variables, identified with backward elimination. The third model had all the significant variables together and others were removed. ANOVA, crosstabs and Dunnet’s test in SPSS version 24 was used to examine the differences in addressing national policies and guidelines and in competence between the different occupational statuses. The alpha level was set at *p* < 0.05.

## Results

### Sample characteristics

The total number of respondents was 2103 with a response rate of 38%. Table 1 shows the sample characteristics. The respondents were mainly practical nurses (75%). Practical nurses have a vocational qualification in health care (a 3-year education). Their role is to provide daily care and they can also deliver medication [24]. Registered nurses or other professionals with bachelor’s degree (such as physiotherapists and occupational therapists) accounted for 16% of the respondents. Moreover, 9% of the respondents reported to have a supervisory position (head nurses and highest level supervisors combined). Some of the respondents’ were manually corrected into one of the groups, if they had described occupation other than named in the list of occupations. Assistants and others, with such an occupational status that could not be included into the three groups, were excluded (*n* = 106). They were excluded due to the fact that they may not participate to a direct care work and in most work units they are not included in the care staff. This left the total sample size of 1997. Most of the respondents were females (92.4%) aged between 19 to 75 years (mean 43.2). On average, they had 8 years of work experience (SD 7.9). The mean grade for quality of care (QoC) provided in the work unit was 8.14 (SD 0.97). This would be referred as ‘good’ on the assessment scale from 4 to 10 [23] (Table 1).

**Table 1:** Sample characteristics

Gender	N (%)					
Female	1845 (92.4)^a^					
Male	87 (4.4)^a^					
Age (yrs)						
Range	19–75					
Mean	43.2					
Occupational status	N (%)					
Practical nurse (PN)	1499 (75.1)					
Registered nurse (RN) or other bachelor level education	314 (15.7)					
Head nurse, supervisor (HN)	184 (9.2)					
QoC mean (SD)^b^	8.14 (0.97)					
Self-asessed professional competence good in						
	%^d^	PN^d^	RN^d^	HN^d^	Non-institutionalized care^d^	Institutionalized care^d^
Pain treatment	63,7	61,6	70,9	68,5	56,2	64,4
SSDMD^c^	62,0	61,6	60,5	67,0	58,0	62,9
Enhancing rehabilitation	70,1	69,7	68,5	76,2	67,2	70,1
Nutrition	67,9	69,0	63,1	67,0	67,3	67,5
Falls prevention	63,8	65,2	63,0	54,1	61,5	64,3
Palliative/end of life care	47,7	44,7	54,5	61,1	32,6	53,0
Multidimensional assessment of function	39,7	37,3	48,1	45,1	39,6	39,4
Pharmacological treatment	58,7	55,0	71,4	67,2	58,9	56,3
Have you at your workplace addressed	Yes (%)	PN^e^	RN^e^	HN^e^	Non-institutionalized care^e^	Institutionalized care^e^
The Act for elderly care?	61,7	60,5	56,3	80,3	57,5	64,4
Quality recommendations?	56,9	57,6	43,5	73,2	48,7	61,1
National Memory policy?	19,3	19,7	14,8	23,6	14,2	22,1
Development program for informal care?	13,8	14,2	10,9	15,5	12,2	14,8
Prevention of home and leisure accident injuries?	17,8	17,6	17,9	19,1	20,3	17,3

^a^Percentage of those who responded the question

^b^Quality of care provided in the work unit on a scale from 4 (the worst) to 10 (the best)

^c^Supporting the self-determination of a person with memory disorders

^d^Percentage of those who assessed their competence ‘good’

^e^Percentage of those who had addressed the guideline or policy

### Multilevel analysis

The results of the multilevel analysis are presented in a Table 2. The null model (model 1) shows that variation in quality of care (QoC) was statistically significantly different between work units (ρ = .14, *p* = 0.00). The ρ-value of .14 indicated that the work unit explained 14% of the variation in QoC. In model 2, where age, occupational status, unit type and the most significant competence variables were included, age was seen to be significantly associated with the variance in QoC (*p* = 0.00), with the effect size (coefficient) value of .07 indicating that a 10 year increase in age increased the grade given to QoC with .07. Furthermore, occupational status was seen to be associated with the variance in QoC. Compared to the reference group (practical nurses), those who had a supervisory position rated the quality on average 0.28 points higher compared to practical nurses (*p* = 0.00). Furthermore, unit type was significantly associated with variance in quality; compared to institutionalized care, those worked in non-institutionalized care (mostly home care workers) had .33 points lower grade for QoC (*p* = 0.00). Of the competence variables, supporting self-determination of a person with memory disorder, nutrition and falls prevention were associated with the variance in QoC (p = 0.00, =0.01, =0.02, and coef. -.11, −.11 and − .11, respectively). This means that if the value for competence in, for example, falls prevention is 1 unit higher (and therefore the competence is lower, since 1 = good), the grade given to QoC is on average − .11 points lower. The next model (model 3) included age, occupational status, unit type and the most significant addressing national guidelines and policies variables. In this model the act for elderly care, quality recommendations and memory policy were significantly associated with variance in QoC (*p* = 0.00, =0.02 and = 0.00, and coef. .17, .13 and .19, respectively). Therefore, for instance, those who had addressed the act for elderly care had on average .17 points higher value for QoC. Supervisory position and unit type were still seen to be significantly associated with the variance in QoC. The last model (model 4) had all the variables that were significant in the previous models. When these variables were tested in the same model, nutrition and quality recommendations did not have an independent contribution anymore, in other words, they lost their significance.

**Table 2:** Multilevel analysis of associations between different areas of self-assessed competence, addressing national guidelines/policies and quality of care provided

	Model 1		Model 2		Model 3		Model 4	
	Null Model		+ all the competence variables		+ all addressing guidelines/policies variables		+ all variables that were significant in previous ones	
Quality of Care^a^	Coefficient (SE)	p*	Coefficient (SE)	p*	Coefficient (SE)	p*	Coefficient (SE)	p*
	8.16 (.03)	**0.00**						
Age			.07 (.02)	**0.00**	.05 (.02)	**0.01**	.06 (.02)	**0.00**
Occupational status								
Head nurses			.28 (.08)	**0.00**	.20 (.08)	**0.01**	.22 (.08)	**0.01**
Nurses			−.10 (.06)	0.11	−.07 (.06)	0.29	*−*.07 (.06)	0.29
Practical nurses			*Reference*		*Reference*		*Reference*	
Unit type								
Non-institutionalized care			−.33	**0.00**	−.33	**0.00**	−.31	**0.00**
Institutionalized care			*Reference*		*Reference*		*Reference*	
Pain treatment			−.05 (.04)	0.18				
SSDMD^b^			−.11 (.04)	**0.00**			−.10 (.04)	**0.01**
Enhancing rehabilitation			−.04 (.05)	0.38				
Nutrition			−.11 (.04)	**0.01**			−.08 (.04)	0.09
Falls prevention			−.11 (.04)	**0.02**			−.10 (.04)	**0.03**
Palliative/end of life care			−.01 (.04)	0.73				
Multidimensional assessment of function			.01 (.04)	0.85				
Pharmacological treatment			.02 (.04)	0.63				
The Act for Elderly Care					.17 (.06)	**0.00**	.16 (.06)	**0.01**
Quality recommendations for elderly care					.13 (.06)	**0.02**	.11 (.06)	0.06
National memory policy					.19 (.06)	**0.00**	.17 (.06)	**0.01**
Prevention of accidents^c^					−.014 (.09)	0.87		
Development program for informal care					−.00 (.07)	0.97		
Rho-value	.14		.15		.13		.13	

*p-values that are significant at a < 0.05 level are bolded

^a^Quality of care provided

^b^Supporting self-determination of a person with memory disorder

^c^Target Programme for the Prevention of Home and Leisure Accidents Injuries

### Differences between occupational statuses

Competence in enhancing rehabilitation was assessed highest among all participants. Results from ANOVA showed that nurses and those with supervisory position assessed their competence better in three out of eight areas compared to practical nurses (*p* < 0.05). However, practical nurses assessed the competence to be better in falls prevention compared to those with supervisory position (*p* < 0.01). The act for elderly care was addressed the most among all participants. Those with supervisory position had addressed the act for elderly care and quality recommendations more than practical nurses (*p* < 0.00). Moreover, nurses had addressed the quality recommendations less compared to practical nurses (p < 0.00). In general, the act for elderly care was addressed the most and development program for informal care the least among all three professional groups.

## Discussion

The aim of this study was to see whether higher self-assessment of own professional competence and addressing national guidelines and policies at workplace are associated with higher self-rated quality of care (QoC) provided in the work unit. Further, this study aimed to examine how commonly different guidelines and policies are addressed, and whether there are differences in competence and addressing policies and guidelines between different professional groups.

Of the eight competence variables, only two seemed to explain the variation in QoC. When considering the findings of this study, a link between the higher rated QoC and higher competence in memory disorders can be seen. Both, competence in supporting the self-determination of a person with memory disorders (SSDMD) and national memory policy were associated with higher rated QoC. With better competence in memory disorders, also better care outcomes could be achieved. And further, with better care outcomes, such as less daily pain, quality of life of a person with memory disorder could be improved like study from Bökberg et al. suggested [25]. Moreover, competence has been seen to be associated with job satisfaction; those nurse assistants who perceived their competence excellent or good in dementia care were more satisfied with their job [26].

Even though clinical guidelines are important and Finnish health care workers seem to have a positive attitude towards them [19], this study demonstrated that they are not addressed very widely. An interesting finding was, however, that an association between addressing the national level guidelines and quality of care provided was found. Therefore, the knowledge regarding the guidelines should be noted at work places, especially amongst supervisors who are the key persons when it comes to addressing the guidelines. In this study we did not examine the reasons why the guidelines are not addressed, still attention should be paid on making sure that there is for example sufficient amount of time for a worker to familiarize oneself with the guidelines and policies. In Canadian context, resource and time constraints, including high patient to nurse ratio and heavy workloads, were recognized as barriers for implementing guidelines [20]. Similar issues, like time constraints, could be the reasons why the workers are not able to address the guidelines in the first place. In this study those with supervisory position had addressed the guidelines more. There have been similar findings; in a study from Kang (2015) those nurses who had a master’s degree had lower perceptions of barriers related to research utilization compared to those with lower education [27]. In the present study, supervisors had presumably more education and most likely a master’s degree. Addressing and covering the guidelines can be seen as a supervisors’ responsibility. Therefore, if they are not widely addressed, issues regarding the management could be further examined.

Age was seen to be associated with the variance in quality and in our study older workers rated the QoC slightly better compared to younger workers. However, the effect size of age was quite low and therefore its contribution was not very large. Previous studies have shown that more experienced and older workers assess their competence higher [13, 28]. It is possible that also in this study older workers rated their competence higher, and therefore age had a contribution on the variance in quality. The type of work unit was also shown to explain the variance in quality. Non-institutionalized care was seen to have lower grade for QoC and this is probably largely explained by home care. In the Finnish home care there has been an increase in the number of clients but not in the number of workers. Workers have been shown to experience time pressure [29] and the stress levels have increased in ten years [30]. Moreover, in Finland time pressure and number of clients in shift are the highest in Nordic countries [30].

The relationship between quality of care and things influencing it seems complex; for example, relevant training was seen to improve the staff well-being and the quality of care. Moreover, role clarity and social support were important factors that influenced the quality of care positively [13] or as Leggat, et al. [31] demonstrated in their study, management and feeling of empowerment were associated with higher QoC. Our study demonstrated that none of the variables we included in the model had a very large independent contribution in explaining the variation in QoC. Therefore, we could state that the variation seems to be more between individuals instead of between units or different forms of care, and that other variables should have been included in the model. Furthermore, work environment and staffing level, might affect the grade of QoC. Previous studies have demonstrated that if the nurse-patient ratio was not experienced to be sufficient, the quality of care was also rated to be worse, compared to higher nurse-patient ratios [32–34]. When taking into account the current state of Finnish elderly care where the amount of patients increase but the amount of staff might not be sufficient, this could possibly be the reason behind the findings of the present study. The characteristics of the clients, that the care is provided for, is also an important factor that can influence the grade given to QoC. The situation might be particularly problematic in home care where workers might consider the clients to be too frail to live at home. With constant rush and worry over clients abilities to cope at home before the next worker visits, the QoC can be rated worse. There are also other studies done in elderly care giving similar results, and showing the problematic situation of workers in home care, that support the results of the present study [30].

### Limitations

There were some limitations in this study. Firstly, the study only examined the relationship of variables related to competence, addressing guidelines and QoC. Higher rated competence in some areas and addressing some of the guidelines were associated with higher QoC but none of the variables had a very large independent contribution, leaving a major part of the variation unexplained. Therefore, as mentioned earlier, other variables should have been included in the analysis to better investigate the factors influencing QoC. For instance, feeling of having to hurry while working and lack of job control could have been important reasons behind the findings of this study. Moreover, this study did not investigate why the individuals in the same work units have differing views on QoC. Secondly, the response rate of the survey was fairly low. This, however, has seen to be a global issue since the web-based surveys’ response rates have declined and they have been shown to be lower than other forms of surveys [35–37]. In this study reasons for non-response could have been, for instance, that the survey was send to work emails and due to high staff turnover not all the emails may have been valid. Another important reason for non-response might be not having enough time during work day. The lack of supervisor support or encouragement on answering the surveys could also explain the non-response or that there are simultaneously many surveys that workers are required to answer. However, the survey’s sample could be considered fairly representative since practical nurses are the largest single occupational group in the Finnish elderly care services, representing approximately 45% of the elderly care staff [38]. Thirdly, since the number of participants was fairly limited, the results of the study could most likely be only generalized to other elderly care units in Finland, however with caution. Due to a cross-sectional nature of this study, no cause and effect relationship could be determined based on the results.

## Conclusions

This study demonstrated that better competence in supporting the self-determination of a person with memory disorders, falls prevention and addressing the act for elderly care and memory policy were associated with higher self-rated QoC. Furthermore, those who were older, had a supervisory position and worked in institutionalized care rate QoC higher. Supervisors’ role can be seen important in securing the addressment of the guidelines, so that the workers are aware of them and are able to change their practices accordingly. However, this study demonstrated that guidelines are not very widely addressed amongst different professionals in the Finnish elderly care. To better investigate things influencing quality of care, other variables should have been examined. In the future, studies should focus on investigating why the differences between individuals in the same work unit are significant and the role of the management.

## Abbreviations

QoC: Quality of care
SSDMD: Supporting self-determination of a person with memory disorder
